# Potentially Modifiable Predictors for Renal Replacement Therapy in Patients with Cardiac Surgery Associated-Acute Kidney Injury: a Propensity Score-Matched Case-Control Study

**DOI:** 10.21470/1678-9741-2018-0206

**Published:** 2019

**Authors:** Wuhua Jiang, Bo Shen, Yimei Wang, Jiarui Xu, Zhe Luo, Xiaoqiang Ding, Jie Teng

**Affiliations:** 1 Department of Nephrology, Zhongshan Hospital, Shanghai Medical College, Fudan University, Shanghai, China.; 2 Shanghai Institute of Kidney and Dialysis, Shanghai, China.; 3 Shanghai Medical Center of Kidney, Shanghai, China.; 4 Department of Cardiac Surgery Intensive Care Unit, Zhongshan Hospital, Shanghai Medical College, Fudan University, Shanghai, China.

**Keywords:** Cardiovascular Surgical Procedures, Acute Kidney Injury, Renal Replacement Therapy

## Abstract

**Objective:**

To discover potentially modifiable perioperative predictors for renal replacement therapy (RRT) in patients with cardiac surgery-associated acute kidney injury (CSA-AKI).

**Methods:**

A cohort of 1773 consecutive cardiac surgery patients with postoperative acute kidney injury (AKI) from January 2013 to December 2015 were included retrospectively. AKI was defined according to the Kidney Disease: Improving Global Outcomes (KDIGO) criteria. The primary outcome was CSA-AKI requiring renal replacement therapy (AKI-RRT). The initiation of RRT was based on clinical judgment regarding severe volume overload, metabolic abnormality (*e.g*., acidosis, hyperkalemia), and oliguria. Patients with AKI-RRT were matched 1:1 with patients without AKI-RRT by a propensity score, to exclude the influence of patients' demographics, comorbidities, and baseline renal function. Multivariable regression was performed to identify the predictors in the matched sample.

**Results:**

AKI-RRT occurred in 4.4% of the entire cohort (n=78/1773), with 28.2% of in-hospital mortality (n=22/78). With the propensity score, 78 pairs of patients were matched 1:1 and the variables found to be predictors of AKI-RRT included the contrast exposure within 3 days before surgery (odds ratio [OR]=2.932), central venous pressure (CVP) >10 mmHg on intensive care unit (ICU) admission (OR=1.646 per mmHg increase), and erythrocyte transfusions on the 1^st^ day of surgery (OR=1.742 per unit increase).

**Conclusion:**

AKI-RRT is associated with high mortality. The potentially modifiable predictors found in this study require concern and interventions to prevent CSA-AKI patients from worsening prognosis.

**Table:** 

Abbreviations, acronyms & symbols				
AKI	= Acute kidney injury		eGFR	= Estimated glomerular filtration rate
AUC	= Area under the curve		GEDVI	= Global end-diastolic volume index
CABG	= Coronary artery bypass grafting		ICU	= Intensive care unit
CI	= Confidence interval		IQR	= Interquartile range
CIN	= Contrast-induced nephropathy		KIDGO	= Kidney Disease: Improving Global Outcomes
CKD	= Chronic kidney disease		LCOS	= Low cardiac output syndrome
CKD-EPI	= Chronic Kidney Disease Epidemiology Collaboration		NYHA	= New York Heart Association
CM	= Contrast media		OR	= Odds ratio
CPB	= Cardiopulmonary bypass		RRT	= Renal replacement therapy
CSA-AKI	= Cardiac surgery-associated acute kidney injury		SCr	= Serum creatinine
CVP	= Central venous pressure		SD	= Standard deviation
DM	= Diabetes mellitus		USD	= United States Dollar

## INTRODUCTION

Acute kidney injury (AKI) is one of the prevalent complications after cardiac surgery, with reported incidences over 30%, while mortality increases fourfold and even a slight renal function decrease can influence short- and long-term survival rates after cardiac surgery^[1]^. However, the patients prone to develop AKI or severe AKI can be possibly identified. There have been several studies^[2-4]^ identifying predictors for cardiac surgery-associated acute kidney injury (CSA-AKI) or AKI renal replacement therapy (RRT), including age, gender, preoperative renal function dysfunction, and comorbidities (*e.g*. hypertension, diabetes mellitus), whereas most of the predictors are unmodifiable and their clinical significance are limited. Recently, several modifiable risk factors have been reported participating in the pathophysiology of CSA-AKI or AKI-RRT^[5-9]^. If analyzed along with unmodifiable predictors, the significance of the modifiable predictors can be concealed due to their lower occurrence, but their association with worsening renal function will be useful for prevention, even if AKI will develop inevitably.

The purpose of this study is to identify the potentially modifiable predictors for AKI-RRT in CSA-AKI patients, with patients' demographics, preoperative comorbidities, and preoperative renal function being controlled utilizing propensity score match.

## METHODS

### Patient Sample

**Fig. 1:**
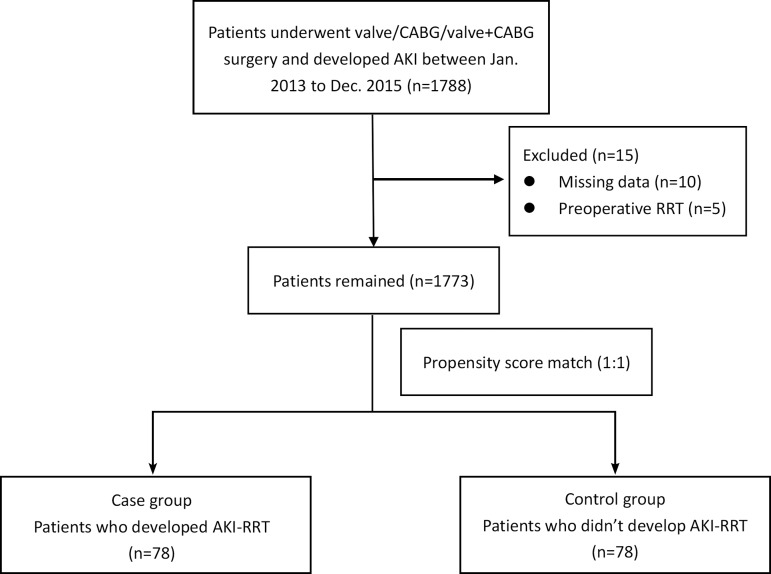
Flow diagram outlining the inclusion and exclusion criteria and study design. AKI=acute kidney injury; CABG=coronary artery bypass grafting; RRT=renal replacement therapy

Given the retrospective design of this case-control study, the ethical board approval from Zhongshan Hospital waived the requirement for informed consent (Approval No. B2017-039). Data from consecutive patients aged 18 years and older who underwent specific cardiac surgery and developed CSA-AKI from January 2013 to December 2015 at Zhongshan Hospital were included in the present study. Inclusion procedures were coronary artery bypass grafting (CABG), valve surgery, or valve combined with CABG surgery. Patients with previous heart transplantation, preoperative mechanical ventilation or tracheotomy, preoperative defibrillator or ventricular assist devices, preoperative RRT, preoperative liver dysfunction, or sepsis were excluded (Figure 1).

A propensity score match (1:1) was performed between AKI-RRT patients and non-AKI-RRT patients to exclude the confounding impact of demographics, preoperative medical status, and baseline renal function. Covariates included age, gender, hypertension comorbidities, diabetes mellitus, preoperative estimated glomerular filtration rate (eGFR), preoperative New York Heart Association (NYHA) classification, and history of contrast media exposure within 2 weeks before surgery.

### Data Collection

All perioperative data were collected and extracted retrospectively from the Zhongshan Hospital's cardiac surgery database. All data were checked twice by professional personnel before being admitted to the database. Demographic and perioperative variables known to be related to AKI-RRT were included in this study after literature review. They included gender, age, comorbidities, contrast media exposure history, preoperative cardiac function status (NYHA classification), baseline eGFR (calculated with Chronic Kidney Disease Epidemiology Collaboration [CKD-EPI] formulae)^[10]^, surgery-related factors (procedures, urgency, cardiopulmonary bypass [CPB] duration, erythrocyte transfusion), and post-operative characteristics of central venous pressure (CVP) and erythrocyte transfusion. Transfusion was performed according to the clinical guideline, the hemoglobin threshold at which erythrocyte transfusion was considered was 70 g/L during CPB and 80 g/L after CPB^[11]^. If there were >1 cardiac surgery procedures performed during the same hospitalization, only the data on the first surgery were considered. Quality assurance checks of the data revealed a missing data rate of <2%. Patients with missing values for variables used in the multivariable analyses were excluded (<1% of the cases).

AKI was defined according to the KDIGO guideline^[12]^ as any of the following: increase in serum creatinine (SCr) by ≥0.3 mg/dL (≥26.5 µmol/L) within 48 hours; or increase in SCr to ≥1.5 times baseline that is known or presumed to have occurred within the prior 7 days; or urine volume <0.5 mL/kg per hour for 6 hours. The primary outcome was postoperative AKI requiring RRT (intermittent hemodialysis or continuous venovenous hemodiafiltration). Consulting nephrologists made decisions about implementing RRT. Indications for RRT were metabolic abnormalities (acidosis, hyperkalemia), anuria, and fluid overload.

### Statistical Analyses

Statistical analyses were performed by SPSS statistics for Windows (Version 24.0., IBM Corp, Armonk, NY, USA). Continuous variables were expressed as mean±standard deviation (SD) and analyzed by unpaired t-tests, with Welch adjustment when necessary. Continuous variables that violated the normality assumption were expressed as median and 25^th^ to 75^th^ percentiles and analyzed by a Mann-Whitney U test. Categorical variables were expressed as absolute (n) and relative (%) frequencies and were analyzed by the Pearson 2-test or Fisher exact test whenever appropriate. Significant level was considered with *P*<0.05.

Univariate analysis was performed in both entire and matched cohorts to identify the potential risk factors for AKI-RRT. In the 1:1 matched sample, each AKI-RRT patient was matched to a non-AKI-RRT patient. Covariate balance in the matched group was assessed with *t*-test and Pearson 2-test or the Fisher exact test, depending on the property of variables. A stepwise forward logistic regression was performed afterward to identify the risk factors only with variables that were significant on univariate analysis (*P*<0.05). The regression model's goodness of fit was assessed with the Hosmer-Lemeshow test. Before performing a logistic regression analysis, the cut-off point was determined for continuous variables on the receiver operating characteristic curve that had the maximal sum of sensitivity and specificity. The cut-off points for CPB times were determined in the previous study^[3]^. The cut-off point for CVP predicting AKI-RRT was 10 mmHg, with a sensitivity of 68% and a specificity of 60%.

## RESULTS

The incidence of AKI-RRT was 4.4 (78/1773) while the in-hospital mortality of the entire cohort and the RRT cohort was 2.5% (44/1773) and 28.2% (22/78), respectively. The characteristics of both entire and matched cohorts were presented in Table 1. Patients who developed AKI-RRT were associated with significantly lower preoperative eGFR, higher prevalence of diabetes mellitus, contrast exposure 3 days before surgery, more significant proportion of valve & CABG procedure, longer CPB time, higher CVP by ICU admittance, and more erythrocyte transfusion than non-AKI-RRT patients.

**Table 1:** Patients' characteristics and perioperative parameters of the AKI-RRT and non-AKI RRT groups in the entire and matched samples.

	Entire cohort			Matched cohort		
	Non-AKI-RRT (N=1695)	AKI-RRT (N=78)	*P*	Non-AKI-RRT (N=78)	AKI-RRT (N=78)	*P*
**Preoperative**						
Male, n (%)	1104 (65.1)	52 (66.7)	0.809	59 (75.6)	52 (66.7)	0.216
Age, median (IQR), years	61 (54,67)	62 (54,69)	0.217	65 (59,70)	62 (54,69)	0.05
**Kidney function**						
Serum creatinine, median (IQR), mg/dl	0.96 (0.8,1.1)	1.0 (0.8,1.3)	0.152	0.9 (0.8,1.2)	1.0 (0.8,1.3)	0.565
eGFR, mean (SD), ml/min/1.73 m^2^	83.7 (22.8)	76.5 (26.9)	0.017	77.7 (25.3)	76.5 (26.9)	0.774
**Medical history**						
Hypertension, n (%)	666 (32.3)	38 (48.7)	0.096	47 (60.3)	38 (48.7)	0.148
DM, n (%)	241 (14.2)	19 (24.4)	0.013	21 (26.9)	19 (24.4)	0.714
NYHA classification >2, n (%)	1128 (66.6)	54 (69.2)	0.057	45 (57.7)	54 (69.2)	0.135
Interval between CM exposure and surgery <3 days, n (%)	52 (3.1)	26 (33.3)		14 (47.9)	26 (33.3)	
**Laboratory index**						
Hemoglobin, mean (SD), g/dL	13.19 (16.7)	12.89 (19.4)	0.243	12.95 (21.6)	12.89 (19.4)	0.675
Albumin, mean (SD), g/L	39.7 (3.4)	38.5 (3.6)	0.688	39.7 (3.3)	38.5 (3.6)	0.481
**Intraoperative**						
Valve, n (%)	1132 (66.8)	49 (62.8)	0.468	30 (38.5)	49 (62.8)	0.002
CABG, n (%)	417 (24.6)	11 (14.1)	0.034	31 (39.7)	11 (14.1)	<0.001
Valve & CABG, n (%)	146 (8.6)	18 (23.1)	<0.001	17 (21.8)	18 (23.1)	0.848
CPB time, mean (SD), min	107.7 (38.6)	138.6 (52.7)	<0.001	111.7 (45.4)	138.6 (52.7)	0.006
**Postoperative**						
CVP, median (IQR), mmHg	8 (6,10)	9 (8,11)	<0.001	8 (7,10)	9 (8,11)	<0.001
*Erythrocyte transfusion, mean (SD)	1.1 (1.6)	5.1 (4.4)	<0.001	0.8 (1.6)	5.1 (4.4)	<0.001
**Prognosis**						
**Renal function complete recovery, n (%)	605 (35.7)	13 (16.7)	<0.001	20 (25.6)	13 (16.7)	<0.001
In-hospital mortality, n (%)	22 (1.3)	22 (28.2)	<0.001	2 (2.6)	22 (28.2)	<0.001
Length of hospital stay, median (IQR), days	13(11,19)	23 (11,47)	<0.001	15 (11,19)	23 (11,47)	0.008
In-hospital expense, median (IQR), USD	18522 (15347,22649)	34712 (23354,45716)	<0.001	14672 (12498,19320)	34712 (23354,45716)	<0.001

AKI=acute kidney injury; CABG=coronary artery bypass grafting; CM=contrast media; CPB=cardiopulmonary bypass; CVP=central venous pressure by intensive care unit admittance; DM=diabetes mellitus; eGFR=estimated glomerular filtration rate, calculated by Chronic Kidney Disease Epidemiology Collaboration (CKD-EPI) formulae; IQR=interquartile range; NYHA=New York Heart Association; RRT=renal replacement therapy; SD=standard deviation; USD=United States Dollar The values are expressed as median (IQR), mean (SD), or number (%). *P*-values are the results of unpaired t-test or Mann-Whitney U test for continuous variables, and χ^2^ test or Fisher's exact test for categorical variables.

* The amount of erythrocyte transfusion refers to the amount of transfusion during both intraoperative and postoperative days of surgery.

** The complete recovery of renal function is defined as a return of creatinine to no higher than baseline and no dialysis.

**Fig. 2:**
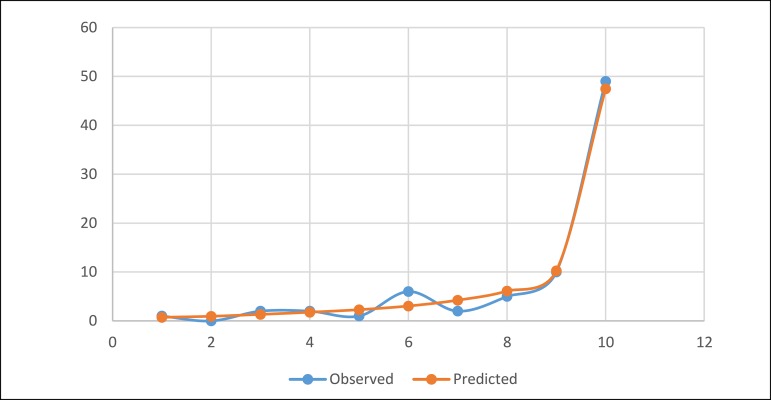
The logistic regression's goodness of fit in the propensity score-match model. χ^2^=7.43, P=0.386

The matched cohort included 78 pairs of patients with and without AKI-RRT. The logistic regression's goodness of fit in the propensity score-matched model was assessed fair with the Hosmer-Lemeshow test (χ^2^=7.43, *P*=0.386; Figure 2). As illustrated by the *P* value of the unpaired *t*-test or Mann-Whitney test, the groups were well-balanced for the variables, which were used for covariates to the propensity score. There were no differences in demographic data, medical history, or preoperative renal function in the matched sample set.

**Fig. 3:**
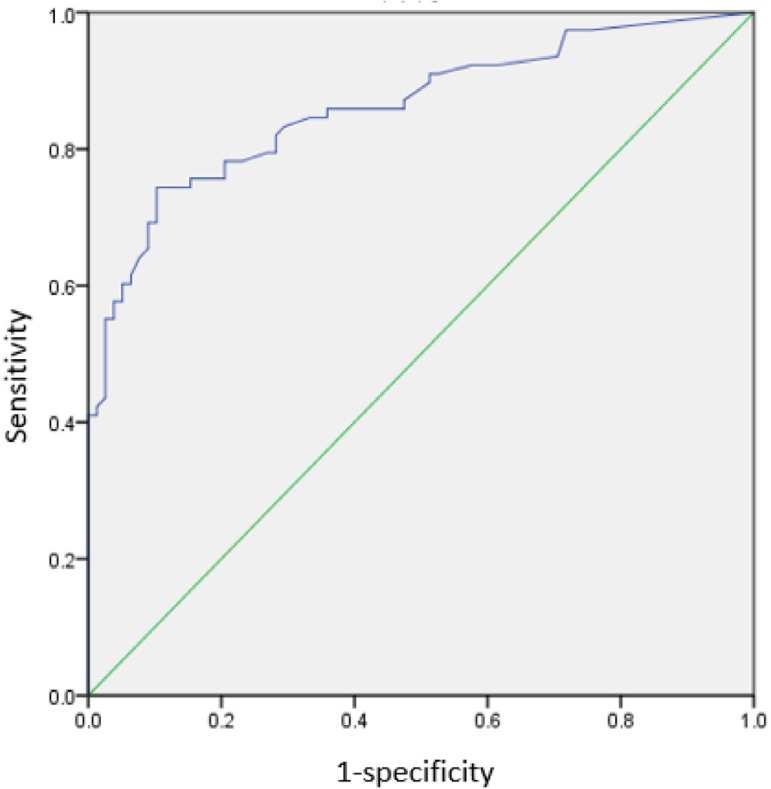
Discrimination of the logistic regression model for the risk of acute kidney injury renal replacement therapy (AKI-RRT) in the matched cohort.

**Fig. 4:**
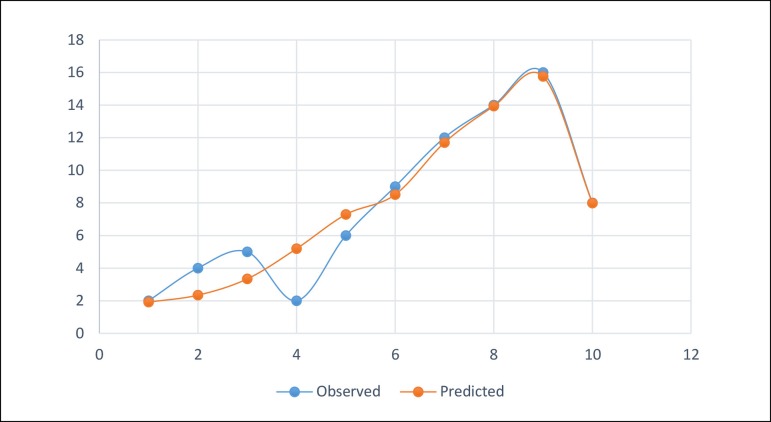
The calibration of the logistic regression model for predicting cardiac surgery-associated acute kidney injury (CSA-AKI) in the matched cohort.

The result of multivariate logistic regression for AKI-RRT prediction in the matched cohorts was presented in Table 2. Resulted predictors for AKI-RRT were the exposure of contrast within 3 days before surgery (odds ratio [OR]=2.932), ICU admission CVP >10 mmHg (OR=1.646 per mmHg increase), and erythrocyte transfusions on the 1^st^ day of surgery (OR=1.742 per unit increase). Discrimination and calibration of the logistic regression model were assessed with receiving operating characteristics curve (Figure 3) (area under the curve [AUC]=0.861) and Hosmer-Lemeshow test (Figure 4) (χ^2^=6.08, *P*=0.638), which indicated an adequate result. AKI-RRT was related to higher in-hospital mortality, longer length of stay, and higher in-hospital expense as well (Table 1).

**Table 2:** Multivariate analysis of risk factors for the development of acute kidney injury in the matched sample.

Covariate	β-coefficient	Odds ratio	95%CI	*P*
Interval between preoperative contrast media exposure <3 days	1.076	2.932	0.951-9.039	0.048
ICU admission CVP >10 mmHg (per mmHg increase)	0.498	1.646	1.156-2.344	0.006
Erythrocyte transfusions (per unit increase)*	0.555	1.742	1.358-2.234	<0.001

CI=confidence interval; CVP=central venous pressure; ICU=intensive care unit; LCOS=low cardiac output syndrome

* The amount of erythrocyte transfusion refers to the amount of transfusion during both intraoperative and postoperative days of surgery.

## DISCUSSION

Although cardiac surgery is booming in developing countries like China, India, and Brazil, the management of complications, including CSA-AKI, requires more concern. However, pharmacologic treatment for CSA-AKI remains controversial^[13]^ and stronger evidence is necessary. To cope with the CSA-AKI global burden, the identification of risk factors for CSA-AKI or AKI-RRT has become the priority.

In many cases, AKI happens inevitably, and the following strategy may encompass AKI's management, including potential RRT protocol rather than medications^[14]^. Similar to previous studies^[15-17]^, the mortality, length of stay, and in-hospital expense of patients who developed AKI-RRT were high in this cohort. Therefore, identifying the risk of AKI-RRT has become a cardinal problem, which is related to consulting nephrologists and the following scheme.

There have been many observational studies focused on the identification of risk factors in the last decade^[2-4]^. These risk factors can be classified as preoperative, intraoperative, and postoperative. Common risk factors include male gender, advanced age, and comorbidities (*e.g*. chronic kidney disease [CKD], diabetes mellitus, hypertension, and cardiac dysfunction). But these risk factors are readily intuited and non-modifiable and their clinical significance are limited. Moreover, it's possible that these non-modifiable risk factors could be concealing the modifiable risk factors. We hypothesized some potentially modifiable predictors from previous studies^[3,5,6,8,9]^ and attenuated the influence of the confounding patient's demographics and baseline clinical parameters using a propensity score-matched case-control study.

Coronary angiography is one of the common preoperative examinations. This procedure aims to screen the patients with high risk of coronary artery disease and provide clinicians with multiple cardiac surgery procedure alternatives. A precise diagnostic method as it is, though, angiography may be risky to susceptible patients due to contrast-induced nephropathy (CIN). The expected increase of SCr appears within 48 hours after contrast media exposure, reaching a peak within the following 5 days. Increased morbidity, hospital stay, and mortality are often associated with CIN^[18]^. Few studies were performed to reveal the relationship between contrast media and CSA-AKI. Not only the incidence of CSA-AKI but both short- and long-term mortalities as well were found higher in patients who experienced CIN than in those who did not^[6]^. Ranucci et al. ^[19]^ found out that surgery on the same day of angiography significantly increases the risk of AKI. In our study, the interval between contrast media exposure and cardiac surgery was investigated, and the cut-off point, which was determined on the receiver operating characteristic curve that had the maximal sum of sensitivity and specificity, was 3 days. This result was also significant both in the entire and the matched cohorts. After the logistic regression, the contrast exposure within 3 days before surgery was one of the predictors for AKI-RRT. As a modifiable predictor, the interval needs lengthening, which may reduce the risk of AKI.

CVP, the downstream pressure of the systemic venous system, is frequently measured in patients undergoing cardiac surgery. Most of the existing literature focused on the accuracy of CVP reflecting the right ventricular end-diastolic pressure. Recently, the importance and role of CVP as a prognostic indicator have also been studied^[20]^. Palomba et al.^[3]^ revealed that the post-operative CVP was one of the predictors for CSA-AKI. In our study, ICU admission CVP >10 mmHg was one of the risk factors for AKI-RRT. Increased CVP implies high renal afterload, while cardiac function recovery delays and the renal perfusion decreases, which may result in AKI. If available, global end-diastolic volume index (GEDVI) shall be monitored simultaneously to evaluate the hemodynamics and renal perfusion. If GEDVI did not increases following CVP, clinicians should be aware of heart compliance reduction by fluid overload. Hemodynamic parameters, including CVP, are helpful to evaluate both patients' condition and previous therapy effect.

Kidneys receive approximately 20% of the cardiac output and they are highly susceptible to circulation hypoxia^[21]^. Acute and numerous blood loss is common during cardiac surgery, and low oxygen delivery is recognized as a risk factor for developing perioperative AKI. Blood transfusions are frequently performed to improve oxygen delivery to the kidneys and other vital organs during cardiac surgery and subsequent ICU stay for ischemic injury prevention. However, previous studies also indicated a correlation between erythrocyte transfusion and CSA-AKI^[22,23]^. The potential harm of erythrocyte transfusion resulted from both patients and blood storage. Patients who received a massive amount of erythrocyte transfusion tended to be in a more critical state and to be more susceptible to hypoxia and CSA-AKI. Besides, it has been reported that the post-transfusion recovery of red blood cells is lower after a long time of blood storage^[24]^. The morphological and biochemical changes during storage are participating in promoting a pro-inflammatory state, impairing tissue oxygen delivery, and exacerbating tissue oxidative stress due to free iron, which is an AKI risk factor for susceptible CPB patients^[23]^. In our study, the preoperative hemoglobin level between AKI-RRT patients and non-AKI-RRT patients was not significant in both the entire and matched cohorts, which indicated a minor relationship between preoperative hemoglobin and post-operative AKI-RRT. On the other hand, the erythrocyte transfusion resulted in a modifiable predictor for AKI-RRT in CSA-AKI patients, and the risk increased along with the amount of transfusion. Therefore, optimizing the transfusion strategy shall be considered in the upcoming clinical trials. Several available therapeutic options to reduce the harm of erythrocyte on CSA-AKI patients were studied^[23,25]^. Interventions reducing perioperative hemodilution by minimizing fluid administration and retrograde autologous priming of the cardiopulmonary circuit, salvage of shed blood, and tolerance of moderate hemodilution can be easily applied.

There are limitations in this study. Firstly, CVP and the erythrocyte transfusion performed with strict indication were not modifiable, but they can be surrogate markers, indicating pathophysiological status, which can be optimized before their onset. Secondly, CSA-AKI was not identified with CIN in patients who underwent catheterization before surgery because both pathophysiological courses overlap. However, a short interval between catheterization and surgery shall not be encouraged yet. Thirdly, this study derived from a single-center retrospective observational study, and prospective randomized clinical trials are needed to verify whether the modification of these modifiable risk factors can genuinely reduce the incidence of AKI-RRT and improve prognosis.

## CONCLUSION

In this study, we attempted to find modifiable risk factors in CSA-AKI patients and aimed to prevent these patients from a continuous worsening renal function. To exclude the influence of the confounder, we performed a propensity score match, and several modifiable risk factors, including contrast exposure within 3 days before surgery, erythrocyte transfusion, and ICU admission CVP, resulted to be the predictors for AKI-RRT.

**Table:** 

Authors' roles & responsibilities	
WJ	Analyzed the data, interpreted the results and writing; final approval of the version to be published
BS	Analyzed the data; interpreted the results and writing; final approval of the version to be published
YW	Participated in data collection and maintenance; final approval of the version to be published
JX	Analyzed the data; final approval of the version to be published
ZL	Participated in reviewing the manuscript; final approval of the version to be published
XD	Designed and directed the study; final approval of the version to be published
JT	Designed and directed the study; final approval of the version to be published
